# Neoadjuvant chemotherapy in relation to long-term mortality in individuals cured of gastric adenocarcinoma

**DOI:** 10.1007/s10120-024-01558-7

**Published:** 2024-10-10

**Authors:** Wilhelm Leijonmarck, Fredrik Mattsson, Jesper Lagergren

**Affiliations:** 1 Upper Gastrointestinal Surgery, Department of Molecular Medicine and Surgery, Karolinska Institutet, Retzius Street 13A, 4th Floor, 171 77 Stockholm, Sweden; 2 School of Cancer and Pharmacological Sciences, King’s College London, London, UK

**Keywords:** Stomach neoplasms, Cancer survivors, Neoadjuvant therapy, Registers, Sweden / epidemiology

## Abstract

**Background:**

Late effects of chemotherapy could affect mortality amongst cancer survivors. This study aimed to clarify if neoadjuvant chemotherapy for gastric adenocarcinoma influences the long-term survival in individuals cured of this tumour.

**Methods:**

This was a nationwide and population-based cohort study that included all individuals who underwent gastrectomy for gastric adenocarcinoma in Sweden between 2006 and 2015 and survived for ≥ 5 years after surgery. The cohort was followed up until death or end of study period (31 December 2020). Multivariable Cox proportional hazards regression was used to provide hazard ratios (HR) with 95% confidence intervals (CI). The HR were adjusted for age, sex, comorbidity, education, calendar year, tumour sub-location, in-hospital complications, and splenectomy. Data came from medical records and nationwide registers.

**Results:**

Amongst 613 gastric adenocarcinoma survivors, neoadjuvant chemotherapy (used in 269 patients; 43.9%) was associated with a decreased crude mortality rate (HR 0.66, 95% CI 0.46–0.96). However, the association attenuated and became statistically non-significant after adjustment for all confounders (HR 0.83, 95% CI 0.56–1.23) and after adjustments solely for age and comorbidity (HR 0.82, 95% CI 0.56–1.20). Stratified analyses did not reveal any statistically significant associations between neoadjuvant chemotherapy and long-term mortality in categories of age, sex, comorbidity, calendar year and tumour sub-location.

**Conclusion:**

Neoadjuvant chemotherapy did not decrease the long-term survival amongst gastric adenocarcinoma survivors. Patients who received neoadjuvant chemotherapy were a selected group characterised by younger age and fewer severe comorbidities and therefore with better chances of long-term survival.

## Introduction

Gastric adenocarcinoma is one of the most common cancers globally and holds a poor prognosis [1, 2]. The mainstay curative treatment is surgical removal with gastrectomy, often combined with perioperative or adjuvant chemotherapy [2]. Tumour recurrences usually occur early after treatment of gastric adenocarcinoma, and patients who survive for 5 years are typically cured [3, 4]. The addition of perioperative and adjuvant chemotherapy to gastrectomy improves cancer survival [2], but there is limited understanding if chemotherapy has any long-lasting or late effects on mortality in survivors of gastric adenocarcinoma.

A recent study from our research group compared the survival amongst gastric adenocarcinoma survivors with the background population of the same age, sex, and calendar year, and observed better survival in patients who had received neoadjuvant chemotherapy in a subgroup analysis [5]. That finding was unexpected and must be examined in a separate study using another design. We hypothesised that the decreased crude mortality rate observed was attributed to a selection process, where patients who underwent neoadjuvant chemotherapy had better overall health and fitness.

This study aimed to clarify if neoadjuvant chemotherapy influences the long-term all-cause mortality in gastric adenocarcinoma survivors.

## Methods

### Design

This nationwide and population-based cohort study included all patients who underwent gastrectomy for gastric adenocarcinoma in Sweden from 2006 to 2015 and survived for at least 5 years. These participants were considered cured of the cancer. The follow-up started 5 years after surgery and ended at death or the end of the study period (December 31, 2020). Data sources were medical records and nationwide registers. The study was approved by the Ethical Review Board in Stockholm, Sweden (2017/141–31/2), and registered at clinicaltrials.gov (identification code NCT05540119).

### Study cohort

The source cohort is the *Swedish Gastric Cancer Surgery Study* (SWEGASS), which includes at least 98% of all patients who underwent gastrectomy for gastric adenocarcinoma between January 1, 2006 and December 31, 2015 in Sweden. A detailed description of SWEGASS has been published previously [6]. In brief, patients with gastric adenocarcinoma who underwent gastrectomy were identified using well-validated nationwide Swedish registers for cancer (*Swedish Cancer Registry* [7]) and inpatient and outpatient care (*Swedish Patient Register* [8]). The final cohort was selected after a review of surgical charts, pathology reports, discharge summaries, and reports from multidisciplinary meetings. Patients in SWEGASS who survived for at least 5 years after gastrectomy were eligible for the present study. We excluded patients older than 78 years at the date of gastrectomy because it was very rare that these patients received neoadjuvant chemotherapy.

### Exposure

The study exposure was neoadjuvant chemotherapy. Patients were considered exposed regardless of whether they discontinued the treatment or reduced the dose due to side effects, corresponding to the intention-to-treat strategy. Patients who did not receive neoadjuvant chemotherapy were categorised as unexposed (reference group). Data on neoadjuvant chemotherapy were collected from the medical records.

### Outcome

The study outcome was death due to any cause, i.e. all-cause mortality. Mortality data came from the *Swedish Cause of Death Register*, which has 100% completeness for the date of death and > 96% completeness for causes of death, including deaths of Swedish citizens who die abroad [9].

### Confounders

Eight variables were considered potential confounders, which are defined and categorised as follows: age (at the start of follow-up, continuous variable), sex (male or female), comorbidity (0, I, or ≥ II, according to the most well-validated version of the Charlson comorbidity index scoring system [10, 11], not counting the gastric adenocarcinoma), education (≤ 9, 10–12, or ≥ 13 years of formal education), calendar year period (at the start of follow-up, 2011–2015 or 2016–2020), in-hospital postoperative complications (0, I–II, or III–IV, according to the Clavien–Dindo classification of surgical complications [12]), tumour sub-location (non-cardia or cardia) and splenectomy (during the gastrectomy, no or yes). Data sources for these variables were medical records, the *Longitudinal Integration Database for Health Insurance and Labour Market* [13], and the *Swedish Patient Register* [8].

### Statistical analysis

Survival curves were depicted using the Kaplan–Meier estimator. Cox proportional hazards regression was used to calculate hazard ratios (HR) with 95% confidence intervals (CI). Four models were analysed: (1) a crude model without any adjustments, (2) a full model with adjustment for all eight potential confounders presented above (‘Confounders’), (3) a model with adjustment for age and comorbidity, and 4) a model with adjustment for six confounders excluding adjustment for age and comorbidity. To evaluate whether any associations between neoadjuvant chemotherapy and long-term mortality were modified by age, sex, comorbidity, calendar year and tumour sub-location, an interaction term was included in the main multivariable model one by one where HR were derived within each stratum. These variables were also categorised as stated above (‘Confounders’), except for age, which was categorised into two groups divided by the median. The proportional hazards assumption was evaluated by log–log survival plots and by calculating the correlations between Schoenfeld residuals for a particular covariate and ranking of individual failure time. The correlations were low and the statistical test was not statistically significant at a level of 0.05 for each variable, indicating that the proportional hazards assumption was met for all variables. We noted that the neoadjuvant group tended to be right censored earlier than the non-neoadjuvant group because patients receiving neoadjuvant therapy underwent gastrectomy in more recent calendar years. Thus, we assessed if the results were robust in a sensitivity analysis where the main analysis approach was replicated in a subgroup of patients with the possibility for a complete 5 year follow-up and where the outcome was 5 year survival. In a second sensitivity analysis, we excluded participants who, according to the *Swedish Patient Register* and the *Cause of Death Register*, had tumour recurrence and died due to gastric adenocarcinoma more than 5 years after gastrectomy. Partial missing data were low, no more than 4.1% for neoadjuvant chemotherapy status or any of the eight potential confounders combined. The analyses were, therefore, managed by complete case analysis, i.e. exclusion of patients without complete data on all variables included in the analysis. The data management and statistical analyses were conducted according to a pre-defined study protocol and were conducted by the first author (WL) under the supervision of a senior biostatistician (FM). The statistical software was SAS version 9.4 (SAS Institute Inc., Cary, NC, USA).

## Results

### Gastric adenocarcinoma survivors

Amongst 768 individuals who survived 5 years after gastrectomy for gastric adenocarcinoma, we excluded 127 due to age older than 78 years, and another 28 due to missing data. Thus, the final study cohort included 613 gastric adenocarcinoma survivors. Table 1 displays the characteristics of the survivors, categorised according to whether they had neoadjuvant chemotherapy or not. The individuals in the neoadjuvant group were younger, had fewer severe comorbidities, had a higher level of formal education, underwent gastrectomy in more recent calendar years and more often had cardia tumours and underwent splenectomy. There were no major differences in the distribution of sex, in-hospital complications or pathological tumour stage between the groups.

**Table 1:** Characteristics of 613 gastric adenocarcinoma survivors who underwent gastrectomy in Sweden from 2006 to 2015, stratified by neoadjuvant chemotherapy

	Neoadjuvant chemotherapy	
	No	Yes
Number of patients (%)	344 (56.1)	269 (43.9)
Age (at the start of follow-up). median (quartile 1;3)	74 (65;79)	69 (61;74)
Sex		
Male	194 (56.4)	157 (58.4)
Female	150 (43.6)	112 (41.6)
Comorbidity (Charlson comorbidity index)		
0	196 (57.0)	166 (61.7)
I	98 (28.5)	87 (32.3)
≥ II	50 (14.5)	16 (5.9)
Education (years)		
≤ 9	124 (36.0)	88 (32.7)
10–12	158 (45.9)	113 (42.0)
≥ 13	62 (18.0)	68 (25.3)
Calendar period (at start of follow-up)		
2011–2015	208 (60.5)	97 (36.1)
2016–2020	136 (39.5)	172 (63.9)
Tumour sub-location		
Non-cardia	316 (91.9)	226 (84.0)
Cardia	28 (8.1)	43 (16.0)
In-hospital complications (Clavien–Dindo score)		
None	229 (66.6)	172 (63.9)
I–II	73 (21.2)	53 (19.7)
III–IV	42 (12.2)	44 (16.4)
Splenectomy		
No	310 (90.1)	223 (82.9)
Yes	34 (9.9)	46 (17.1)
Pathological tumour stage		
0–I	180 (52.3)	130 (48.3)
II	107 (31.1)	85 (31.6)
III–IV	55 (16.0)	53 (19.7)
Missing	2 (0.6)	1 (0.4)

### Neoadjuvant chemotherapy and mortality in gastric adenocarcinoma survivors

**Fig. 1:**
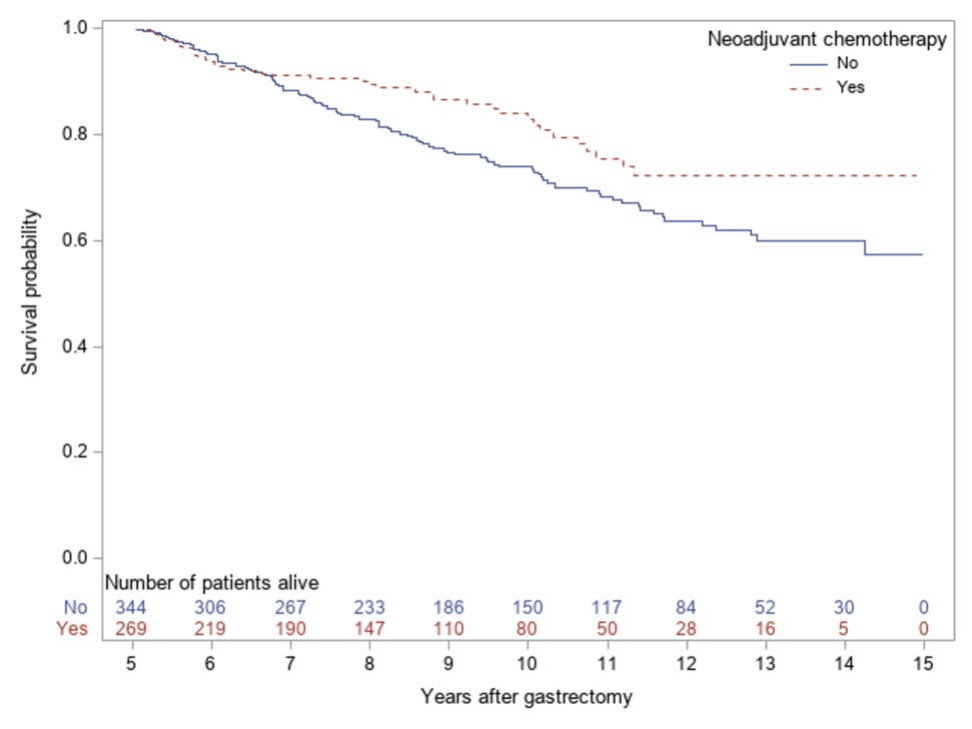
Kaplan–Meier survival curves amongst 613 gastric adenocarcinoma survivors, starting after 5 years of gastrectomy, stratified by neoadjuvant chemotherapy

The crude analysis showed a decreased mortality rate amongst gastric adenocarcinoma survivors who had received neoadjuvant chemotherapy compared to those in the non-neoadjuvant group (HR 0.66, 95% CI 0.46–0.96, Table 2, Fig. 1). The association attenuated and became statistically non-significant after adjustment for all confounders (HR 0.83, 95% CI 0.56–1.23) and after adjustment for only age and comorbidity (HR 0.82, 95% CI 0.56–1.20) (Table 2). The crude point estimate did not change when adjustment was made for the six confounders but not for age or comorbidity (HR 0.66, 0.45–0.97, Table 2). The results were consistent in the sensitivity analyses of survivors with at least 5 years of follow-up (fully adjusted HR 0.72, 95% CI 0.40–1.27, *n* = 305), and when individuals with death due to tumour recurrences (*n* = 29; 4.7%) were excluded (fully adjusted HR 0.87, 95% CI 0.56–1.36, *n* = 584).

**Table 2:** Neoadjuvant chemotherapy and mortality amongst gastric adenocarcinoma survivors, presented as hazard ratios (HR) with 95% confidence intervals (CI)

	Neoadjuvant chemotherapy	
	No	Yes
Crude HR (95% CI)	1.00 (reference)	0.66 (0.46- 0.96)
HR (95% CI)*	1.00 (reference)	0.83 (0.56–1.23)
HR (95% CI)**	1.00 (reference)	0.82 (0.56–1.20)
HR (95% CI)***	1.00 (reference)	0.66 (0.45–0.97)

*Adjusted for age, sex, comorbidity, education, calendar year, tumour sub-location, in-hospital complications and splenectomy

**Adjusted for age and comorbidity

***Adjusted for sex, education, calendar year, tumour sub-location, in-hospital complications and splenectomy, but not for age or comorbidity

The stratified analyses did not reveal any statistically significant adjusted HR comparing neoadjuvant chemotherapy and risk of mortality in sub-categories of age, sex, comorbidity, calendar year or tumour sub-location, although the point estimate for women who underwent neoadjuvant chemotherapy was lower than for men (Table 3).

**Table 3:** Neoadjuvant chemotherapy and mortality amongst gastric adenocarcinoma survivors stratified by covariates

	Neoadjuvant chemotherapy	
	No	Yes
	Adjusted hazard ratio (95% confidence intervals)*	
Age (at the start of follow-up)		
≤ 71	1.00 (reference)	0.68 (0.39–1.19)
> 71	1.00 (reference)	0.87 (0.51–1.47)
Sex		
Male	1.00 (reference)	1.06 (0.66–1.68)
Female	1.00 (reference)	0.52 (0.26–1.04)
Comorbidity (Charlson comorbidity index)		
0	1.00 (reference)	0.78 (0.46–1.33)
I	1.00 (reference)	0.88 (0.48–1.62)
≥ II	1.00 (reference)	0.93 (0.30–2.89)
Calendar period (at start of follow-up)		
2011–2015	1.00 (reference)	0.79 (0.49–1.26)
2016–2020	1.00 (reference)	0.94 (0.48–1.87)
Tumour sub-location		
Non-cardia	1.00 (reference)	0.89 (0.58–1.35)
Cardia	1.00 (reference)	0.60 (0.24–1.54)

*Adjusted for age, sex, comorbidity, education, calendar year, tumour sub-location, in-hospital complications and splenectomy

## Discussion

This study found no support for the hypothesis that neoadjuvant chemotherapy influences long-term all-cause mortality in gastric adenocarcinoma survivors. Neoadjuvant chemotherapy was associated with a decreased crude mortality rate, but this association attenuated and became statistically non-significant after adjustment for confounders, particularly age and comorbidity.

Amongst methodological strengths of this study is the nationwide and population-based design with a high participation rate, which provided a large and unselected cohort. The extensive collection and review of medical records and the complete and well-maintained nationwide registries available in Sweden enabled accurate information on neoadjuvant chemotherapy, all-cause mortality, and confounders, and removed the otherwise common issue of losses to follow-up. Amongst weaknesses is the lack of data on specific types of chemotherapy. However, the mortality risk after neoadjuvant chemotherapy did not change over time when stratified by calendar years. Therefore, any changes in chemotherapy treatment regimens during the study period did not appear to impact mortality amongst gastric adenocarcinoma survivors. Information on adjuvant treatment was unavailable. However, chemoradiotherapy was not commonly used in Sweden during the study period. Amongst weaknesses is also the inherent risk of residual confounding, e.g. by lifestyle factors (tobacco smoking, alcohol overconsumption, obesity) and performance status. The limited number of patients remaining many years after gastrectomy reduced the statistical power, especially for the later follow-up years and for the separate analysis of women.

The study showed that adjustment for age and comorbidity changed the crude estimates, reflecting that patients who received neoadjuvant chemotherapy were a selected group characterised by younger age and fewer severe comorbidities, in whom the added burden of neoadjuvant treatment was justified [14]. This finding aligns with the study hypothesis. It was more common with higher educational levels, gastrectomy in more recent calendar years, cardia tumours and splenectomy in the neoadjuvant chemotherapy group, but these factors did not influence the long-term survival.

A recent study from our research group compared survival between gastric adenocarcinoma survivors and the corresponding background population and observed better survival amongst gastric adenocarcinoma survivors who underwent neoadjuvant chemotherapy [5]. That study did not have data to adjust for comorbidity, which might have confounded the result. Other studies examining long-term effects of chemotherapy amongst cancer survivors, in general, have observed an accelerated ageing process after chemotherapy with new comorbidities or worsening of existing comorbidities [15], thus decreasing the chances for survival. This is particularly evident in childhood cancer survivors, where an estimated 81% develop a serious chronic health condition before age 45 [15], although this may be difficult to distinguish from the natural ageing process amongst adult cancer survivors [16]. Studies on long-term side effects of chemotherapy amongst gastric adenocarcinoma survivors in particular are scarce. The standard chemotherapy regimen in Sweden during the time of this study was based on the findings from the MAGIC trial [17, 18]. Long-term side effects of this treatment may include sequelae from acute chemotherapy toxicity and heart failure due to epirubicin-induced cardiomyopathy [19]. Yet, the present study indicates that neoadjuvant chemotherapy does not reduce the long-term survival amongst gastric adenocarcinoma survivors. FLOT (fluorouracil plus leucovorin, oxaliplatin, and docetaxel) became the standard regimen for gastric adenocarcinoma in Sweden some years after the treatment period of this study. Given the similar number of severe acute adverse between FLOT and the MAGIC regimen [20] and the absence of epirubicin in FLOT (thus eliminating the risk for epirubicin-induced cardiomyopathy), suggests no increased mortality risk amongst gastric adenocarcinoma survivors who received FLOT compared to the MAGIC regimen. However, future studies are needed to confirm this.

In conclusion, neoadjuvant chemotherapy does not seem to influence the long-term risk of all-cause mortality after having survived gastric adenocarcinoma. The survivors with a history of neoadjuvant chemotherapy were a selected group characterised by younger age and fewer severe comorbidities with better chances for long-term survival. Recognising these differences is important when assessing long-term effects of chemotherapy on mortality amongst survivors of gastric adenocarcinoma.

## Data Availability

Data not available.
